# Detection of common clones of *Salmonella enterica* serotype Infantis from human sources in Tehran hospitals 

**Published:** 2018

**Authors:** Reza Ranjbar, Hedieh Rahmati, Leili Shokoohizadeh

**Affiliations:** 1 *Molecular Biology Research Center* *, Systems Biology and Poisonings Institute* *, Baqiyatallah University of Medical Sciences, Tehran, Iran*; 2 *Department of Microbiology, Faculty of Sciences, Tonekabon Branch, Islamic Azad University, Tonekabon, Iran*; 3 *Department of Microbiology, Faculty of Medicine, Hamadan University of Medical Sciences, Hamadan, Iran *

**Keywords:** *Salmonella* Infantis, Multi Locus Sequence Typing, Iran

## Abstract

**Aim:**

The aims of this study were to investigate antibiotic resistance pattern and molecular characterization of *Salmonella *Infantis strains, isolated from human sources in Tehran hospitals from 2008 to 2010.

**Background:**

Infection caused by Salmonella is one of the major public health problems. Despite the clinical importance of Salmonella enteric subsp. enteric serovar Infantis in humans, there is no information available about the common clones of *Salmonella *Infantis in clinical isolates in Iran.

**Methods:**

*S*. Infantis strains were identified by conventional microbiological and serological testing. The antimicrobial susceptibility of the *S.*Infantis isolates was determined using the disk diffusion method. The genetic relatedness and the dominant clones of *S. *Infantis strains were detected by the Multi Locus Sequence Typing (MLST) and pulsed-field gel electrophoresis (PFGE) techniques.

**Results:**

More than 80% of the *S. *Infantis isolates represented multidrug-resistant patterns. PFGE revealed high genetic similarity among *S. *Infantis strains. While, MLST indicated high-clonal similarity among strains, where all *S. *Infantis strains were assigned to the ST32 sequence type.

**Conclusion:**

This is the first study in Iran conducted to determine the sequence types of *S. *Infantis in clinical isolates using MLST. The genetically closed MDR *S. *Infantis clones were responsible for the apparent endemic occurrence of salmonellosis, caused by this Salmonella serovar, in Tehran.

## Introduction

 Gastrointestinal tract infection is still one of the most serious public health issues in many geographic areas and is endemic in most countries including Iran (1).

*Salmonella* is an important cause of gastrointestinal tract and food-borne infections worldwide. Infections caused by multi-drug resistant *Salmonella *spp. are increasing in many countries, including Iran (2,3).

Most of the *Salmonella* serotypes are potential pathogens for humans and animals. *Salmonella *Typhi, *Salmonella *Infantis, and *Salmonella *Enteritidis are known as the most frequent serovars of *Salmonella* in humans, worldwide (4,5).

*S. *Infantis has been one of the most frequent serovars in many countries, including Asian countries*. S. *Infantis has been isolated from humans, animals and vegetables, meats (e.g. broiler and chicken), *S*. Infantis is more prevalent in poultry than in other animals (5,7).

In Iran, *S. *Infantis has been isolated from broilers and human sources (10, 11,13). Poultry is known as one of the major putative reservoirs for *Salmonella *in Iran. Dissemination of multidrug-resistant (MDR) *S. *Infantis has been reported in Iran and other countries. This has caused problems for the clinical and veterinary sectors (2, 3, 10, 11, 13).

Different molecular typing methods of *S.*Infantis collected from food and clinical sources may increase the understanding of the epidemiology and evolution of *S*. Infantis strains in Iran. An important aspect of molecular typing of bacteria strains is determining the clonal and strain distributions among various environments. Molecular typing methods are proved to be helpful for this purpose. Therefore, several molecular typing methods have been developed to investigate the molecular epidemiology of microbial pathogens (14). Molecular typing approaches such as Multilocus Sequence Typing (MLST), Multiple-Locus Variable number tandem repeat Analysis (MLVA), pulsed-field gel electrophoresis (PFGE), repetitive sequence-based PCR (rep-PCR), ERIC-PCR, and ribotyping have been effectively used in phylogenetic and epidemiological studies of *S. *Infantis. MLST, known as a typing method based on PCR and sequencing, helps to explore the clonal lineages and evolutionary pathways of bacteria (5, 11, 15-19). There is only one instance of common clones of *Salmonella* Typhimurium reported from Iran, which was detected by MLST (20).

There is no phylogenetic study of *S*. Infantis by MLST in Iran. Therefore, there is no information of the sequence types or clones of *S.* Infantis in food or human sources. We aimed to report the common sequence types of *S*. Infantis isolated from clinical samples for the first time in Iran hospitals. The aim of this study is to investigate the antibiotic resistance patterns, genetic linkage and dominant clones of *S. *Infantis strains isolated from human sources in Tehran hospitals which was done by MLST and PFGE. 

## Methods

***Salmonella*** **Infantis Isolates**

The study included all *Salmonella* strains isolated from all cases of enteritis in patients hospitalized in three major hospitals; Baqiyatallah, Mofid Children's Hospital and Children’s Medical Center, in Tehran, during 2008–2010. These strains were isolated from clinical samples, including blood, urine, and stool.* S. *Infantis isolates were identified and confirmed according to the conventional standard of biochemical and serological tests (21).

**Antimicrobial susceptibility test**

Antimicrobial susceptibility of *S. *Infantis to the following was detected, based on the CLSI criteria (22): Ampicillin (AMP 10μg), ceftriaxone (CRO 30μg), ceftazidime (CAZ 30μg), amikacin (AN 30μg), nalidixic acid (NA 30μg), kanamycin (K 30μg), amoxicillin/ clavulanic acid (AMC 20/ 10μg), trimethoprim/ sulfamethoxazole (SXT 1.25/ 23.75μg), streptomycin (S 10μg), tetracycline (TE 30μg), chloramphenicol (CHL 30μg), ciprofloxacin (CIP 5μg), gentamicin (10μg), cefotaxime (CTX 30μg), and imipenem (IPM 10μg) (Mast Company, UK).

**Pulsed-Field gel electrophoresis**

The clonal relatedness of *S*. Infantis isolates was analyzed by the PFGE method with the help of *Xba*I enzyme, using a CHEF-DRIII apparatus (Bio-Rad, USA). It was conducted in accordance with the CDC (Centers for Disease Control and Prevention) Pulse Net protocol (www.cdc.gov/pulse.net). The restriction patterns were compared using Dice-coefficient online program and grouped by the Unweighted Pair-Group Method with Arithmetic Mean (UPGMA). *S*. Infantis isolates were clustered based on the similarity with a coefficient higher than 90%.

**Multilocus sequence typing**

A total of 15 *S*. Infantis strains were selected for the MLST analysis. These isolates were chosen based on PFGE patterns, antibiotic resistance profiles, location of sampling, and type of specimens. *S*. Infantis genomic DNAs were extracted using a commercial extraction kit (CinnaGen, Iran). The internal fragments of seven housekeeping genes; *aro*C, *dna*N, *hem*D, *his*D, *pure*, *suc*A, and *thr*A of *S. enterica *were amplified using specific primers as described in the online MLST database (http://mlst.warwick.ac.uk/mlst/dbs/ Senterica). All the PCR products were subjected to sequencing (Macrogen, South Korea) and the sequencing data were recorded in salmonella MLST database. Phylogenetic analysis was performed by eBURST. 

## Results

***Salmonella*** **I** ***nfantis*** **isolates**

The study included 6050 patients who had been admitted with enteritis symptoms to Baqiyatallah, Mofid, and Children’s Medical Center in Tehran, during 2008–2010. Of these, 110 patients of which 49 (44.5%) were females and 61 (55.5%) were males showed symptoms of salmonellosis. Salmonella infection was diagnosed based on clinical presentations and laboratory confirmation. The majority of the patients (80%) were less than 12 years of age. Most of the *Salmonella* strains (94 isolates) were recovered from stools, whereas the remaining strains were isolated from urine, blood or other biological fluids.

**Figure 1:**
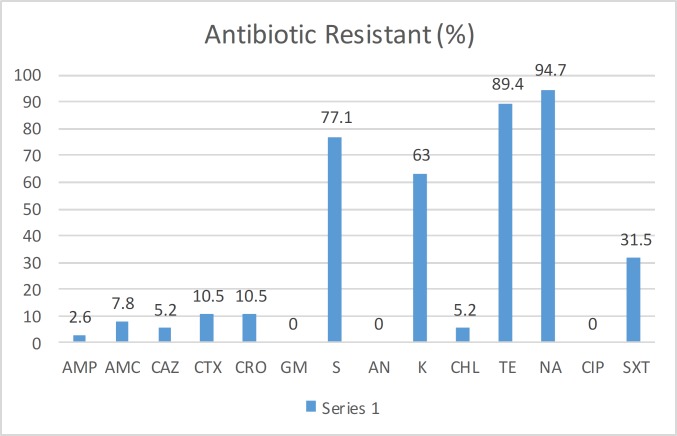
Rate (%) of antibiotic resistance in *Salmonella *Infantis strains isolated from the clinical samples collected from the hospitals in Tehran

**Table 1:** The frequency of antibiotic resistance patterns (%) in *Salmonella* Infantis isolates

Antibiotic resistance patterns	Frequency (%)
SXT/ S/TE/N/CRO/CTX /AM/ CAZ/NA/AM	2 (6.4%)
CF/S/TE/ N/CRO/CTX/ /AM/ CAZ/NA/AM	1 (3.2%)
S/TE/ N/CRO/CTX/ AM /CAZ/NA/AM/K	1(3.2%)
SXT/S/TE /N/NA/K	2(6.4%)
SXT/S/TE /NA/K	1(3.2%)
S/TE/ NA/K	15 (48.3%)
S/TE/ NA	3 (9.6%)
SXT/TE /NA	1(3.2%)
S/TE	1(3.2%)
TE/NA	1(3.2%)
S	3(9.6%)

Of 110 *Salmonella* isolates, 34.5% (38 strains) were identified as *Salmonella *Infantis. Thirty one *S. *Infantis strains (89%) were recovered from pediatric patients under 12 years and seven strains were isolated from patients over 12 years.

**Antimicrobial susceptibility testing**

The results of the antimicrobial susceptibility testing were shown in Figure 1. All the isolates were susceptible to gentamicin, amikacin, cefotaxime, imipenem, and ciprofloxacin. The high-level resistance to nalidixic acid, tetracycline, and kanamycin were detected in 94.7% (n = 36), 89.4% (n = 34), and 63% (n = 24) of the isolates, respectively. Multidrug-resistant (MDR) pattern was detected in 80% (n=25) of the isolates, which showed resistance to three or more antibiotic classes. Simultaneous resistance to streptomycin, tetracycline, nalidixic acid, and kanamycin (S/TE/NA/K) as well as streptomycin, tetracycline, nalidixic acid, kanamycin, ceftriaxone, cefotaxime, ceftazidime, and ampicillin** (**S/TE/D/N/NA/K/CRO/CTX/CAZ/AM) was observed in 45% (n = 14) and 13% (n = 4) of *S. *Infantis isolates, respectively. Antibiotic resistance patterns of isolates are present in Table 1.

**Pulsed-Field gel electrophoresis**

**Figure 2:**
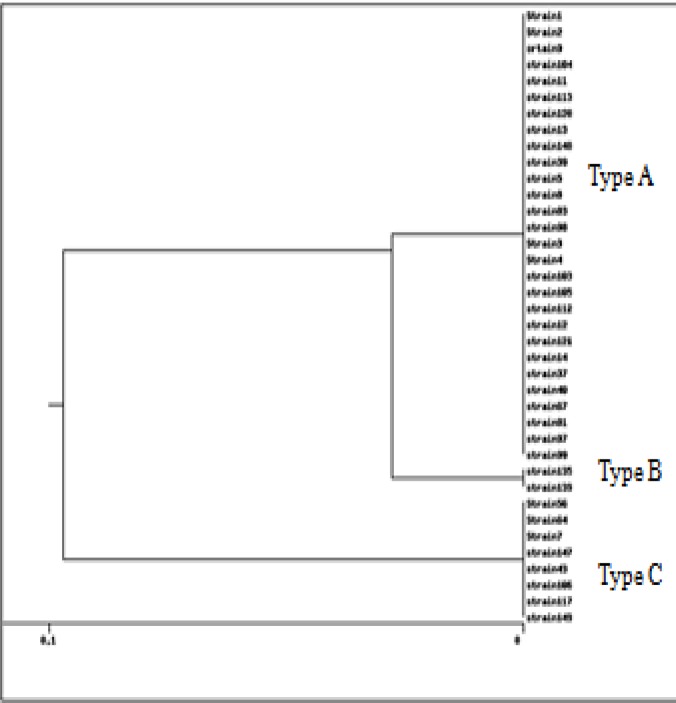
Dendrogram showing genetic relationships among 39 *Salmonella *Infantis strains based on PFGE patterns. The strains were compared using Dice index and clustered by UPGMA method

According to the PFGE analysis, high-level similarity (≥ 90%) was detected among *S. *Infantis strains isolated from Tehran hospitals. The isolates were divided into three different PFGE types or pulsotypes—A, B, and C (as shown in figure 2). The main PFGE profile or pulsotype was type A, which included 28 strains. There was no relationship between antibiotic resistant profiles, type of specimens, location of sampling, and PFGE patterns.

**Multi-Locus sequence typing**

All the *S. *Infantis isolates were assigned to sequence type 32 (ST32), using MLST. The allele profiles of the *aro*C, *dna*N, *hem*D, *his*D, *pure*, *suc*A, and *thr*A genes were 17, 18, 22, 17, 5, 21, and 19 in ST32, respectively. ST32 was located in the eBURST group number 31 (eBG1) or clonal complex 31.

## Discussion

The present study provides evidence of the presence of high frequency of MDR, observed in *S*. Infantis clinical samples collected from the hospitals in Iran. The genetic relationship between these strains was also elucidated by PFGE and MLST techniques.

According to our results, the frequency of *S. *Infantis in human samples was 28%, which is higher than the rates reported earlier from Tehran, Iran by Tajbakhsh *et al*. (8%) and Hamidian *et al*. (5.4%) (10,13). However, Rahamni *et al*. reported higher prevalence (75%) of *S*. Infantis in broiler farms located in three northern provinces of Iran (11).

In Italy, the surveillance system reported that the rate of isolation of *S*. Infantis from human infections ranged from 2-7% between the years 1980 and 2009—several years even represented the third- or fourth-most prevalent serotype (12, 23).

Our results showed high prevalence (80%) of MDR *S*. Infantis isolates, which is higher than the other studies in Iran (10, 13). The high frequency of MDR among *S*. Infantis is in agreement with the results of several studies from different countries such as Japan, Hungary, Italy, Brazil, and Germany. These studies identified healthy poultry as a potential reservoir of *S.* Infantis (9, 14, 15, 24, 25). In a study from Iran, partly similar antibiotic resistant patterns were detected in *S. *Infantis isolated from broilers. All the 27 *S. *Infantis isolates were resistant to ciprofloxacin, nalidixic acid, tetracycline, spectinomycin, streptomycin, and sulfamethoxazole (11). Furthermore, the resistance of these 27 *S.* Infatis to antibiotics was higher than the rates detected and reported by Tajbakhsh *et al*. in the *S. *Infantis isolated from the stool of patients from six hospitals in Iran. However, consistent with our results, they found that the resistance to tetracylin and nalidixic acid was more than the others antibiotics (10).

There is only one published study that used PFGE for molecular typing of *S*. Infantis strains in Iran. Rahmani *et al*. revealed highly similar PFGE patterns in *S*. Infantis strains isolated from poultry, indicating clonal relatedness across different geographical locations in Iran (11). The PFGE results of our study showed a high genotypic similarity among the strains isolated from humans admitted in different hospitals in Tehran, Iran. The PFGE analysis showed that these strains belonged to a uniquely large cluster. PFGE was used successfully for molecular typing of *S. *Infantis strains, isolated from different sources worldwide. Epidemiological evidences confirmed clonal distribution of *S*. Infantis isolates. Dionisi *et al*. showed high similarity among *S. *Infantis isolated from different sources in Italy. Cluster analysis concluded that isolates with same resistance patterns belonged to a large cluster with > 90% genetic similarity (12). Nógrády *et al*. found two large related clusters of *S*. Infantis isolates in various European countries—of which the Austrian and Polish MDR clones of a cluster are identical with, or closely related to, the main Hungarian clone (8). Hauser *et al*. showed that two major closely related genotypes of *S. *Infantis were isolated from broiler, meat, and pork in Germany (26).

Additionally, MLST showed a high clonal similarity among all the strains assigned to the same ST32. This is consistent with the previous results found by others from different sources worldwide (5, 24, and 26-28). Our findings indicated that the majority of *S. *Infantis strains studied may have descended from a common precursor that is responsible for the contamination in humans in Tehran.

The findings of this study provide further information on the molecular epidemiology of *S. *Infantis isolated from human sources in Tehran. The genetically closed MDR *S. *Infantis clones are responsible for the apparent endemic occurrence of salmonellosis, in Tehran. Further molecular epidemiology investigations are required to assess the linkage and clonal relatedness of the *S. *Infantis strains isolated from different sources, such as humans, food and animals, in a different period of time and region in Iran.
